# Endothelial Progenitor and Mesenchymal Stromal Cells in Newborns With Congenital Diaphragmatic Hernia Undergoing Extracorporeal Membrane Oxygenation

**DOI:** 10.3389/fped.2019.00490

**Published:** 2019-11-22

**Authors:** Neysan Rafat, Christian Patry, Ursula Sabet, Tim Viergutz, Christel Weiss, Burkhard Tönshoff, Grietje Beck, Thomas Schaible

**Affiliations:** ^1^Department of Neonatology, University Children's Hospital Mannheim, University of Heidelberg, Mannheim, Germany; ^2^Department of Pediatrics I, University Children's Hospital Heidelberg, Heidelberg, Germany; ^3^Department of Pharmaceutical Sciences, Bahá'í Institute of Higher Education (BIHE), Teheran, Iran; ^4^Department of Anesthesiology and Critical Care Medicine, University Medical Center Mannheim, University of Heidelberg, Mannheim, Germany; ^5^Department for Medical Statistics and Biomathematics, Medical Faculty Mannheim, University of Heidelberg, Mannheim, Germany; ^6^Department of Anesthesiology, Helios Dr. Horst-Schmidt Clinic, Wiesbaden, Germany

**Keywords:** progenitor cells, stem cells, ECMO, congenital diaphragmatic hernia, neonates

## Abstract

**Background:** Endothelial progenitor (EPC) and mesenchymal stromal cells (MSC) can regenerate damaged endothelium and thereby improve pulmonary endothelial dysfunction. We do not know, how extracorporeal membrane oxygenation (ECMO) might affect EPC- and MSC-mediated regenerative pathways in patients with congenital diaphragmatic hernia (CDH). Therefore, we investigated, if ECMO support impacts EPC and MSC numbers in CDH patients.

**Methods:** Peripheral blood mononuclear cells from newborns with ECMO-dependent (*n* = 18) and ECMO-independent CDH (*n* = 12) and from healthy controls (*n* = 12) were isolated. The numbers of EPC and MSC were identified by flowcytometry. Serum levels of vascular endothelial growth factor (VEGF) and angiopoietin (Ang)-2 were determined.

**Results:** EPC and MSC were elevated in newborns with CDH. ECMO-dependent infants had higher EPC subpopulation counts (2,1–7,6-fold) before treatment compared to ECMO-independent infants. In the disease course, EPC and MSC subpopulation counts in ECMO-dependent infants were lower than before ECMO initiation. During ECMO, VEGF serum levels were significantly reduced (by 90.5%) and Ang2 levels significantly increased (by 74.8%).

**Conclusions:** Our data suggest that ECMO might be associated with a rather impaired mobilization of EPC and MSC and with a depression of VEGF serum levels in newborns with CDH.

## Introduction

Severe congenital diaphragmatic hernia (CDH) in newborns is accompanied by pronounced hypoplasia of both pulmonary tissue and the pulmonary vasculature (1). Thus, these patients often develop mechanical ventilator refractory hypoxia and hypercarbia (2). Extracorporeal membrane oxygenation (ECMO) can support gas exchange through the perioperative stage of CDH repair, but neither surgical repair nor ECMO will sufficiently remedy the underlying underdevelopment of lung parenchyma and vasculature (3). Furthermore, ECMO has been shown to induce a systemic inflammatory response characterized by an upregulation of pro-inflammatory cytokines and inflammatory markers and thereby might promote dysfunction in pulmonary endothelium (4, 5).

To improve survival and outcome of severe forms of CDH, therapies are needed that resolve pulmonary hypertension and the underlying hypoplasia of pulmonary tissue, such as therapeutic measures to promote growth of the pulmonary vasculature. In this respect, endothelial progenitor cells (EPC) and mesenchymal stromal cells (MSC) appear to be interesting potential research targets, since they have been demonstrated to promote angiogenesis and endothelial cell regeneration in several diseases with underlying endothelial dysfunction and damage in numerous experimental and pre-clinical studies (6, 7). With regard to CDH, strong evidence exists that both EPC and MSC can promote pulmonary vascular growth and attenuate pulmonary hypertension in the developing lung (8–10). First studies have demonstrated that ECMO might influence the mobilization of EPC and MSC into the systemic circulation (11–13). But there are no studies focusing differentially on the impact of CDH and MSC mobilization and on the underlying molecular mechanisms. As severe forms of CDH will render ECMO support necessary, we need to understand, if ECMO support might influence endogenous EPC- and MSC-mediated pathways underlying both pulmonary vasculature growth and the resolution of pulmonary endothelial dysfunction in those patients.

In this study, we hypothesized that ECMO in newborns with CDH upregulates the mobilization of EPC and MSC as well as serum levels of VEGF and Ang2 as potent progenitor cell mobilizers.

## Methods

### Subjects

Patient recruitment took place between 10/2010 and 04/2016. Newborns with CDH receiving ECMO (*n* = 18) or no ECMO (*n* = 12) were selected from the neonatal intensive care unit (NICU) of the Department of Neonatology of the University Children Hospital Mannheim, University of Heidelberg directly after birth. In our manuscript, patients suffering from severe CDH with the need for and application of ECMO will be referred to as “ECMO-dependent.” Patients suffering from CDH but had not undergone ECMO will be referred to as “ECMO-independent.” The indication for and the allocation to ECMO were performed based on the recommendations made by the CDH EURO Consortium Consensus Team (2015 Update) (2), which are: inability to maintain adequate pre-ductal or post-ductal oxygen saturations, increased PaCO_2_ and respiratory acidosis, increasing peak inspiratory pressure (PIP) count to maintain adequate arterial oxygen saturation, elevated lactate, resistant systemic hypotension, and prolonged oxygenation index of more than 40 for at least 3 h (2). Estimation of CDH disease severity and thus assessment of post-partal ECMO-need was based on prenatal diagnostic measures including fetal ultrasonography for liver position and measurement of observed to expected (O/E) lung to head ratio (LHR) as well as fetal MRI for lung volume (LV) determination in special cases (14). Mortality was defined as death occurring within 28 days after diagnosis. Exclusion criteria were congenital heart defects (except patent ductus arteriosus and persistence of the foramen ovale), inborn errors of metabolism, other anatomical pulmonary anomalies, severe pneumonia/sepsis and pulmonary contusions. None of the study patients developed symptoms suggestive of transfusion associated lung injury following donor blood transfusion. Laboratory findings of each patient were recorded. Blood samples from healthy term newborns (*n* = 12) at day 3 post-partum served as healthy controls. This study was approved by the local ethics committee of the Medical Faculty Mannheim of the University of Heidelberg and informed consent was obtained from the parents of all study subjects.

### Blood Sampling

Blood (2 mL) was obtained from the central venous catheter of ECMO-dependent newborns before connection to the ECMO system (day 0). Cannulation for ECMO in all study patients of the ECMO-dependent group was performed within 24 h after birth. Further blood sampling was performed at day 1, 3, and 7 during ECMO support, as well as directly before cannulation and at day 7 and 14 after decannulation or, respectively, on the day of discharge from the ICU, if discharged earlier than 14 days after decannulation. Blood samples from ECMO-independent newborns were obtained from the central venous catheter at the day of birth (day 0) and on day 3, 7, and 14 or, respectively, on the day of discharge from the ICU, if discharged earlier than 14 days after admission. In healthy controls, 2 mL of blood were collected in tubes containing sodium citrate (0.105 M) as anticoagulant by insertion of a 24-gauge cannula intravenously. All blood samples were processed within 4 h after collection. To reflect the respective course of the disease, we averaged all obtained blood samples for each patient of each group. The data generated by that measure are referred to as “disease-course”-data in the ongoing text. Supplemental Figure S1 provides a flow chart visualizing the experimental design, timing of blood sampling, patient recruitment, and assessment of patient outcome in our study.

### Flow Cytometry

Peripheral blood mononuclear cells (PBMC) were prepared by density gradient centrifugation using Ficoll-Hypaque (Amersham Biosciences, Freiburg, Germany). The expression of cell-surface antigens was determined by immunofluorescence staining as described previously (15). One hundred microliters of PBMC (containing 3 × 10^6^ cells) were incubated with 20 μL of FcR-blocking reagent (Miltenyi Biotec, Bergisch-Gladbach, Germany) for 10 min to inhibit non-specific bindings. Thereafter, the cells were incubated at 4°C for 30 min with either 10 μL of PE-conjugated anti-human CD133 monoclonal antibody (Miltenyi Biotec, Bergisch-Gladbach, Germany), 10 μL of FITC-conjugated anti-human CD34 monoclonal antibody (BD Biosciences, Heidelberg, Germany), 5 μL of PerCP-conjugated anti-human CD45 monoclonal antibody and 10 μL of APC-conjugated anti-human CD31 monoclonal antibody or 5 μL of PE-conjugated anti-human CD90 monoclonal antibody (Miltenyi Biotec, Bergisch-Gladbach, Germany), 5 μL of FITC-conjugated anti-human CD29 monoclonal antibody (BD Biosciences, Heidelberg, Germany), 2 μL of PerCP-conjugated anti-human CD34 monoclonal antibody and 5 μL of APC-conjugated anti-human CD73 monoclonal antibody. Titration experiments have been performed for all antibodies. Isotype-matched immunoglobulin G1 and immunoglobulin G2a antibodies (DakoCytomation, Hamburg, Germany) served as negative controls and were used for each patient and measurement. The cells were washed three times to remove unbound antibodies and were finally resuspended in 400 μL of FACS Cellfix solution (BD Biosciences, Heidelberg, Germany). FACS analysis was performed on a FACSCalibur flow cytometer (BD Biosciences), and the data were analyzed using WinMDI 2.8 software (Scripps Research Institute, La Jolla, CA). A minimum of 500,000 events were collected. FACS analysis of each probe was performed in triplicates. The frequency of the expression of surface antigens was determined by a two-dimensional side-scatter/fluorescence dot-plot analysis of the samples after appropriate gating. EPC subpopulations were defined according to current protocols as CD45^dim^/CD34^+^, CD45^dim^/CD34^+^/CD133^+^, and CD45^dim^/CD34^+^/CD133^+^/CD31^+^ (16, 17) and MSC subpopulations—according to the position statement of the International Society for Cellular Therapy (18)—were defined as CD34^−^/CD29^+^/CD73^+^, CD34^−^/CD29^+^/CD73^+^/CD90^+^, CD34^−^/CD73^+^/CD90^+^, and CD34^−^/CD29^+^/CD90^+^ (19). EPC and MSC subpopulation numbers were expressed as percentage of total PBMC in each patient or control.

### Enzyme-Linked Immunosorbent Assay

The serum concentrations of vascular endothelial growth factor (VEGF) and Angiopoietin 2 (Ang2) were assessed using enzyme-linked immunosorbent assay kits (R&D Systems, Wiesbaden-Nordenstadt, Germany) in triplicate samples obtained from 1 mL of serum. The enzyme-linked immunosorbent assays were performed according to the manufacturer's instructions.

### Statistical Methods

All quantitative data are presented as median (range) and mean ± SD. Both parametric and non-parametric methods were used, as appropriate. All variables were examined for normal and non-Gaussian distribution by the Kolmogorov-Smirnov test. For comparison among normally distributed groups, one-way ANOVA, followed by pairwise multiple comparison (Student-Newman-Keuls method), was used. For non-normally distributed data, the non-parametric Kruskal-Wallis test followed by an all pairwise multiple comparison (Dunn's test) was used. Correlation analyses (according to Pearson or Spearman) were considered for all target variables that were considered statistically significant. *p* < 0.05 was considered statistically significant for all results. All analyses were performed using the SAS system release 9.3 (SAS Institute Inc., NC, USA).

## Results

### General Characteristics of the Study Cohort

Forty-two newborns (19 female and 23 male) were included in our study. The ECMO-dependent group consisted of 18 and the ECMO-independent group of 12 CDH patients. The healthy control group comprised 12 healthy newborns. In total, six patients died, resulting in an overall in-hospital mortality of 20%; five cases of death in the ECMO-dependent group (28%); and one case of death in the ECMO-independent group (8%). For characteristics of the study population, see Supplemental Table S1.

### Laboratory Findings in the Study Cohort

At day 0 there were no significant differences in the basic blood cell counts including total leukocyte count (LC), hemoglobin (Hb), hematocrit (Hct), creatinine (Crea), and c-reactive protein (CRP) between the ECMO-dependent, the ECMO-independent and the healthy control group. The platelet count (Plt) at day 0 was significantly lower in the ECMO-dependent compared to the ECMO-independent group (Table 1). In the disease course, mean serum levels of Crea and CRP were elevated but Plt remained lower in the ECMO-dependent group compared to the ECMO-independent group (Table 1). CRP serum levels in the disease course in the ECMO-dependent group and in the ECMO-independent group were significantly higher compared to the controls, whereas Hb and Hct levels were significantly lower in both CDH groups compared to healthy controls (Table 1). Laboratory findings before (day 0) and directly after (day 1) initiation of ECMO support are displayed in Supplemental Table S2. There were significant changes in Crea, Plt, Hb, and Hct. In addition, LC did drop from day 0 to day 1 in the ECMO-dependent group, but this finding was not significant (*p* = 0,619, Supplemental Table S2).

**Table 1:** Laboratory findings of study subjects.

**Creatinine, blood cell count and c-reactive protein in all study subjects at day 0**			
	**ECMO-dependent**	**ECMO-independent**	**Control**
	**Mean** **±** **SD**	**Mean** **±** **SD**	**Mean** **±** **SD**
Crea [mg/dl]	0.77 ± 0.29	0.73 ± 0.14	0.27 ± 0.00 (*n* = 1)
Hb [g/dl]	14.1 ± 1.36	14.5 ± 1.47	15.9 ± 1.89
Hct [%]	40.4 ± 4.42	40.5 ± 3.65	44.3 ± 6.35
Leukocytes [x10^9^/l]	13.1 ± 5,86	18.4 ± 7.21	10.7 ± 3.84
Platelets [x10^9^/l]	161 ± 51.7^*^	232 ± 65	303 ± 146
CRP [mg/dl]	7.06 ± 15.9	2.21 ± 11.8	0.00 ± 0.00
**Creatinine, blood cell count and c-reactive protein in all study subjects in the disease course**			
	**ECMO-dependent**	**ECMO-independent**	
	**Mean** **±** **SD**	**Mean** **±** **SD**	
Crea [mg/dl]	0.75 ± 0.26^*^	0.46 ± 0.13	
Hb [g/dl]	12.3 ± 0.70^#^	12.7 ± 1.77^#^	
Hct [%]	35.0 ± 1.80^#^	35.7 ± 4.53^#^	
Leukocytes [x10^9^/l]	10.9 ± 4.91	15.1 ± 7.3	
Platelets [x10^9^/l]	111 ± 21.9^*^^#^	302 ± 71.9	
CRP [mg/dl]	24.5 ± 22.3^*^^#^	10.6 ± 16.5^#^	

*Crea, creatinine; CRP, c-reactive protein; Hb, hemoglobin; Hct, hematocrit*.

*Laboratory results of blood cell count, creatinine, and c-reactive protein in all study subjects at day 0 and in the disease course*.

* *Significant difference between ECMO-dependent group and the ECMO-independent group (p < 0.05)*.

# *Significant difference in comparison to the control group (p < 0.05)*.

### Subpopulations of EPC and MSC at Day 0 and in Disease Course

**Figure 1:**
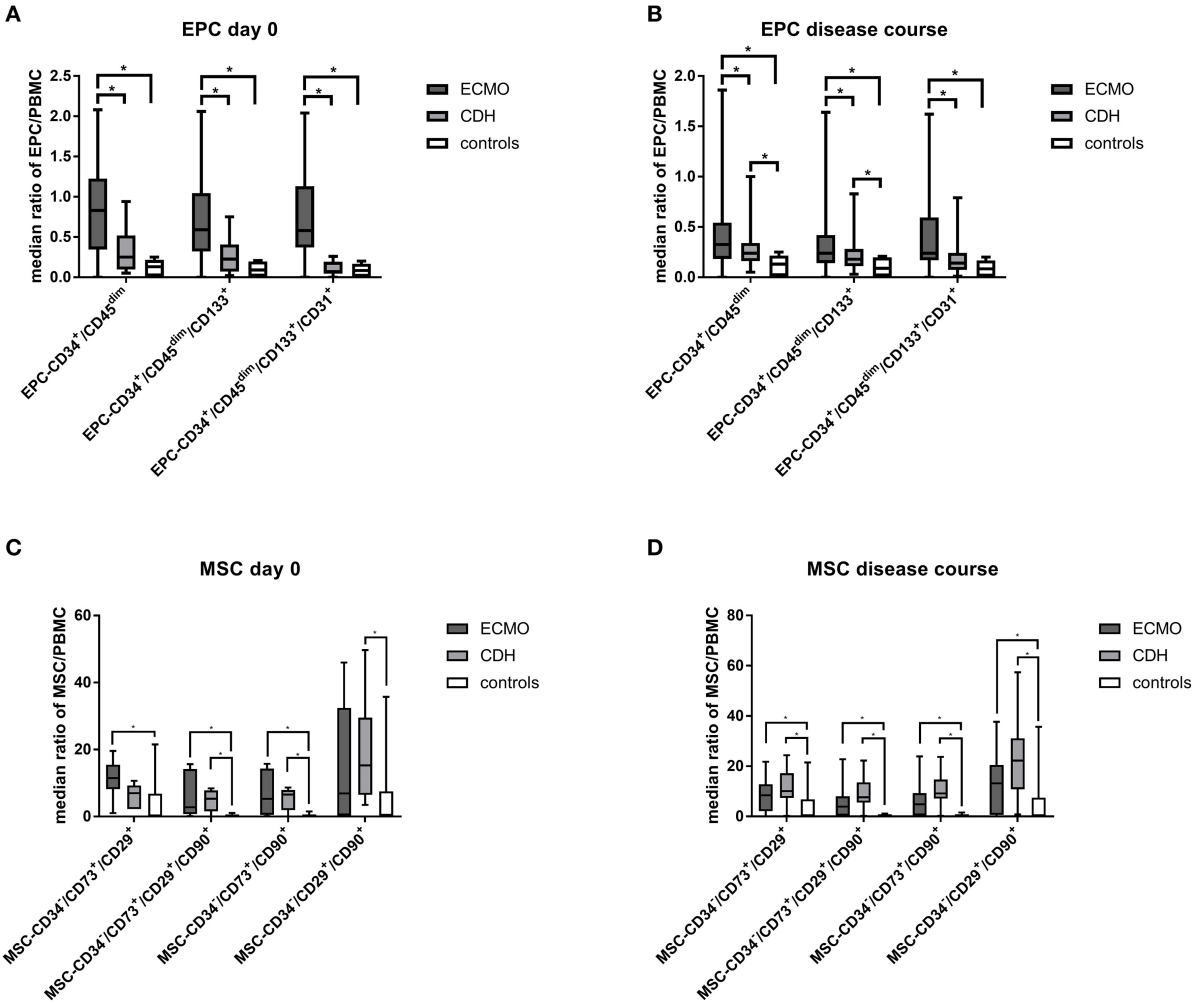
Detection of endothelial progenitor (EPC) and mesenchymal stromal cells (MSC). EPC and MSC subpopulation counts at day 0 **(A,C)** and in the disease course **(B,D)** in the ECMO-dependent (ECMO), the ECMO-independent (CDH), and the healthy control group. ^*^Marks a significant difference (*p* < 0.05).

**Figure 2:**
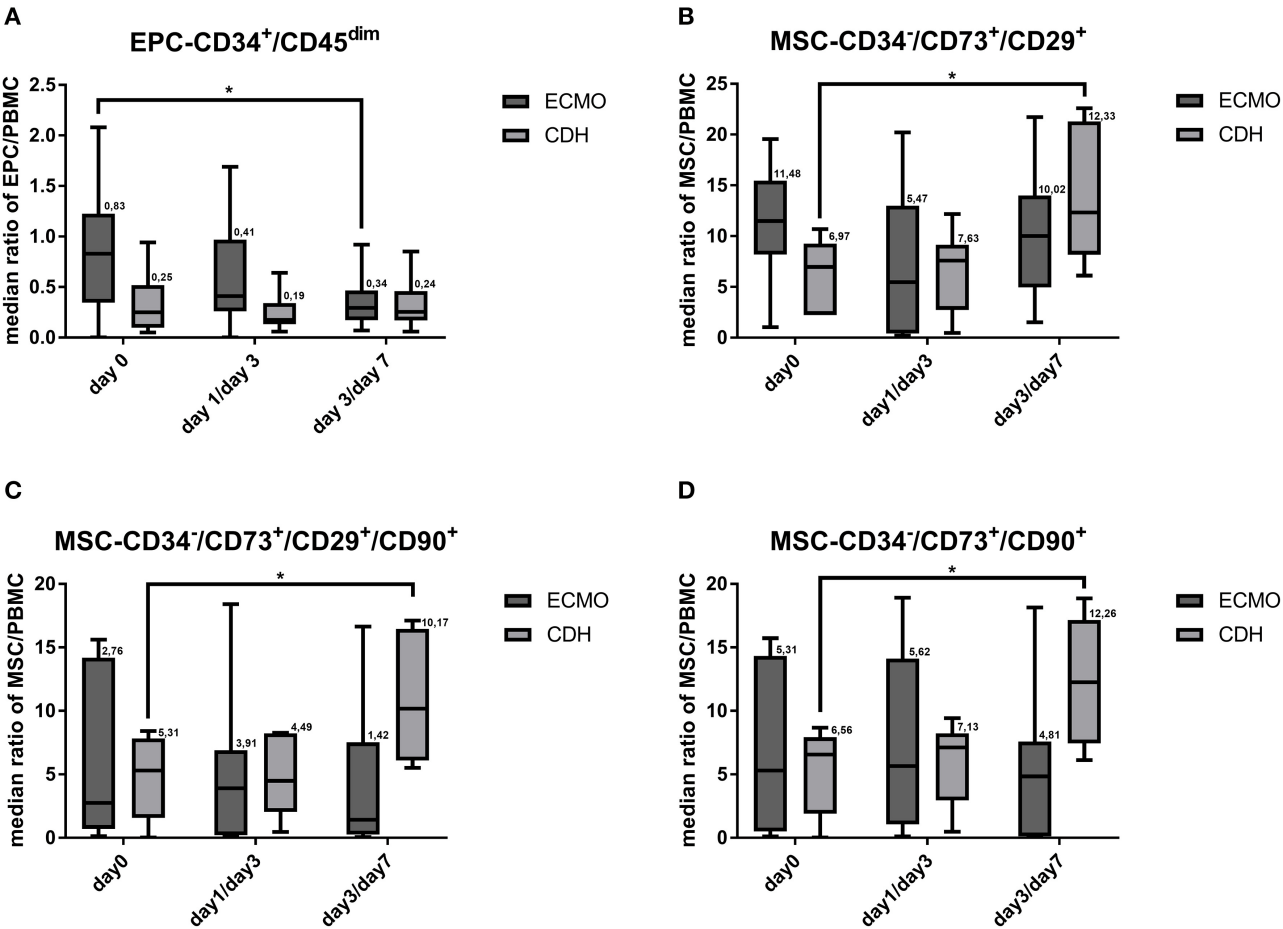
Subpopulations of endothelial progenitor (EPC) and mesenchymal stromal cell (MSC) in the disease course. Counts of the subpopulations EPC-CD45^dim^/CD34^+^ **(A)**, MSC-CD34^−^/CD73^+^/CD29^+^ **(B)**, MSC-CD34^−^/CD73^+^/CD90^+^**(C)**, and MSC-CD34^−^/CD73^+^/CD29^+^/CD90^+^ **(D)** at different times during the disease course (blood samples in the ECMO-dependent group at day 0, day 1, and day 3, blood samples in the ECMO-independent group at day 0, day 3, and day 7). ^*^Marks a significant difference (*p* < 0.05).

At day 0, the counts of all analyzed EPC populations (EPC-CD45^dim^/CD34^+^, EPC-CD45^dim^/CD34^+^/CD133^+^, EPC-CD45^dim^/CD34^+^/CD133^+^/CD31^+^) were significantly higher in the ECMO-dependent group compared to the ECMO-independent group and to the healthy control group (Figure 1, Supplemental Table S3). There were no significant differences in MSC subpopulation counts between both CDH groups at day 0, yet the counts of most MSC subpopulations in the healthy control group were significantly lower compared to both the ECMO-dependent and the ECMO-independent group at day 0 (Figure 1, Supplemental Table S3). In the disease course, mean counts of EPC-CD45^dim^/CD34^+^, EPC-CD45^dim^/CD34^+^/CD133^+^, and EPC-CD45^dim^/CD34^+^/CD133^+^/CD31^+^ remained significantly higher in the ECMO-dependent group compared to the ECMO-independent group (Figure 1, Supplemental Table S3). The MSC subpopulation counts tended to be higher in the ECMO-independent group compared to the ECMO-dependent group, but this was statistically not significant (Figure 1, Supplemental Table S3). However, MSC subpopulation counts were significantly higher in the ECMO-independent group compared to healthy controls (Figure 1, Supplemental Table S3). Furthermore, a comparison of single blood sample results revealed that EPC subpopulation counts were significantly reduced in late disease stages (results shown for EPC-CD45^dim^/CD34^+^) in the ECMO-dependent group, whereas subpopulation counts of MSC did not change significantly (Figure 2). In the disease course of the ECMO-independent group, EPC subpopulation counts did not change significantly but the counts of most analyzed MSC subpopulations were significantly increased in late disease stages (Figure 2).

### Correlation of Cell Populations With Relative Lung-to-Head Ratio and Lung Volume

Results of prenatal CDH diagnostics are shown in Supplemental Tables S5, S6. Relative lung-to-head ratio (rLHR) and relative lung volume were inversely correlated with numbers of EPC-CD45^dim^/CD34^+^/CD133^+^ (rLHR: *r* −0.58, *p* = 0.0044; rel. lung volume: *r* −0.49, *p* = 0.0217; Supplemental Figure S2). There was no other significant correlation.

### Serum Levels of Mobilizing Factors

**Figure 3:**
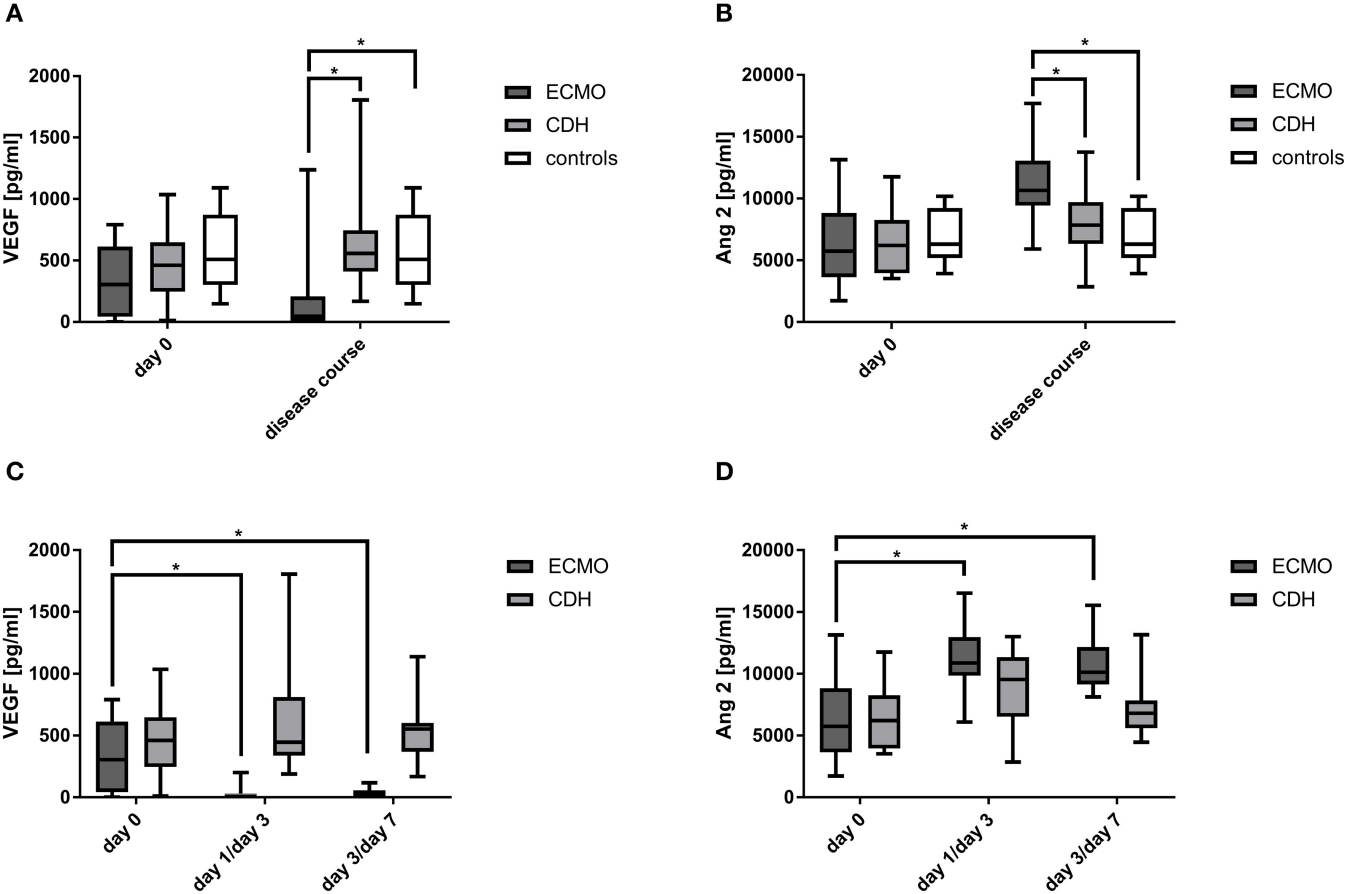
Detection of mobilizing factors in the disease course. Concentrations of vascular endothelial growth factor (VEGF) **(A)** and Angiopoietin 2 (Ang2) **(B)** at day 0 and in the disease course as well as at different time points during the disease course (blood samples in the ECMO-dependent group at day 0, day 1, and day 3, blood samples in the ECMO-independent group at day 0, day 3, and day 7) **(C,D)**. ^*^Marks a significant difference (*p* < 0.05).

At day 0, VEGF serum levels did not differ significantly in all three groups, yet in the disease course VEGF serum levels were significantly reduced in the EMCO-dependent group but remained stable in the ECMO-independent and in the healthy control group (Figure 3, Supplemental Table S4). At day 0, Ang2 serum levels, similar to VEGF, did not differ significantly between both CDH groups and the healthy controls, yet in the disease course Ang2 serum levels were significantly increased in the ECMO-dependent group but remained stable in the ECMO-independent and the healthy control group (Figure 3, Supplemental Table S4). Mean Ang2 serum levels in the disease course were significantly higher in the ECMO-dependent group compared to both the ECMO-independent and the healthy control group (Figure 3, Supplemental Table S3).

### Correlation Between VEGF Serum Levels and EPC CD45^dim^/CD34^+^

Correlation analysis revealed a significant association between decreased VEGF serum levels and EPC CD45^dim^/CD34^+^ counts in the disease course in the ECMO-dependent group (Pears. Corr. Coeff.: 0.65, *p* = 0.012). Furthermore, VEGF serum levels were associated with MSC CD34^−^/CD29^+^/CD73^+^/CD90^+^ counts in the ECMO-dependent group (Pears. Corr. Coeff.: 0.9154, *p* = 0.003).

## Discussion

In this study, we examined the influence of ECMO on the mobilization of EPC and MSC in newborns with CDH. Contrary to our hypothesis, our results indicate, that ECMO support in newborns with CDH is associated with decreased numbers of circulating EPC and MSC. Furthermore, ECMO support in newborns with CDH is associated with decreased VEGF and increased Ang2 serum levels.

The mobilization of EPC and MSC is differentially regulated in several diseases with marked endothelial dysfunction or systemic inflammation such as sepsis and ARDS (15, 20, 21). Animal models of these diseases and experimental studies have demonstrated that EPC and MSC beneficially influence endothelial function and promote regeneration based on proangiogenic signaling (22–25). It seems likely that EPC and MSC might also beneficially influence the endothelial dysfunction present in CDH-associated pulmonary dysplasia (26). First studies investigating EPC counts in CDH patients show conflicting results. Baker et al. found an increase of endothelial colony forming cells (ECFC) in cord blood from 6 term infants born with CDH compared to healthy controls (27). In contradiction, Fujinaga et al. observed a distinct decrease of ECFC colonies in cord blood of full term CDH infants compared to healthy controls (28). Our results are similar to the findings of Baker et al. (27) but we have detected other populations of EPC and applied a different methodology by using flowcytometry and not cell culture assays. Therefore, our findings might not be entirely comparable. We observed a reduction of EPC subpopulation counts, whereas MSC subpopulation counts remained stable in ECMO-dependent newborns with CDH. Interestingly, MSC subpopulation numbers were increased in the disease course of ECMO-independent patients. These data indicate an association between ECMO and the suppression of both EPC and MSC mobilization in newborns with CDH.

Furthermore, our results show that—besides EPC- also mean MSC counts were elevated in CDH patients compared to healthy controls at day 0, when excluding the influence by ECMO. As ECMO seems to be associated with suppressed EPC and MSC mobilization, this could negatively—or at least not beneficially- impact vessel development in the severely impaired pulmonary vessel network in CDH patients undergoing ECMO. This hypothesis is supported by the current literature, which suggests that low numbers of EPC in newborns are associated with endothelial and vascular dysfunction and thereby with the development of bronchopulmonary dysplasia (29, 30). As EPC numbers in the ECMO-dependent group before the start of ECMO and in the early therapy stages were higher compared to the ECMO-independent group, we suggest, that there might be an increased endogenous EPC mobilization which is triggered by intense vascular dysfunction in severe forms of CDH. Higher EPC numbers in these patients might reflect the increased need for vascular regeneration in severe pulmonary vascular hypoplasia. This increased need for vascular regeneration might be interfered with by the observed decrease of EPC numbers. Thus, the observed decrease of EPC numbers in late disease stages during ECMO might interfere with endogenous vascular repair capacity in neonates with severe CDH. In contrast, MSC numbers did not decrease during ECMO-therapy, as EPC numbers did. This finding suggests, that EPC replacement in severe CDH during ECMO might be more beneficial, than MSC replacement. However, due to the regenerative characteristics of EPC and MSC, we hypothesize, that an increase in EPC and MSC counts in CDH patients, could counteract pulmonary endothelial cell dysfunction and improve maturation in CDH induced pulmonary vessel hypoplasia. In line with this, transplantation of MSC has been demonstrated to reduce pulmonary vessel dysplasia in a nitrofen-induced congenital diaphragmatic hernia model in rats (31). A presumable reduction of CDH associated pulmonary vessel dysplasia by an endogenous or exogenous increase of EPC and/or MSC might also beneficially effect ECMO treatment intensity and duration in severe forms of CDH.

When looking at the mobilizing growth factors VEGF and Ang2 in our study, CDH patients had significantly decreased VEGF serum levels during ECMO support, while Ang2 serum levels were significantly increased. The decrease of VEGF was present directly after initiation of ECMO (day 1) and could not be detected in ECMO-independent newborns. In general, VEGF is strongly involved in the mobilization of EPC, promotes EPC- and MSC-dependent angiogenesis and improves MSC viability (32–35). Our data suggest that the decrease of EPC subpopulations and the defective increase in MSC subpopulations observed in our study might be associated with the decrease of VEGF serum levels during ECMO in CDH patients. In support of this, VEGF serum levels correlated significantly with decreased EPC-CD34^+^/CD45^dim^ numbers during ECMO support. A previous study demonstrated elevated VEGF-A serum levels in newborns with CDH compared to normative data available in the literature (36). Contrary to that, VEGF serum levels between ECMO-independent CDH patients and healthy controls did not differ significantly in our study. Healthy control subjects in our study had higher VEGF serum levels compared to the aforementioned normative data, which makes comparison with the above mentioned study difficult. Ang2 promotes EPC migration and adhesion to damaged endothelial layers, mediating endothelial regeneration (37), but its levels are also associated with endothelial barrier breakdown and consecutive vascular leakage in sepsis and settings of acute lung injury (38–40). The increased Ang2 serum levels during ECMO in our study might either mediate and/or result from an intensified vascular permeability due to the ECMO-induced systemic inflammation. Similar findings were observed in adult patients undergoing cardiopulmonary bypass (41).

To interpret the results of our study within a clinical context and with respect to the design, we have to discuss the limitations of our study. Although our study has a prospective design, it is—however—an observational and not an interventional study, which has an impact on gross data interpretation. We were not in control of certain confounders and can mainly observe associations and not deduce cause and effect. Thus, the conclusions drawn from our results on the impact of ECMO on EPC, MSC, VEGF, and Ang2 remain mainly associative. Also, the need for blood products or volume support during ECMO, which we did not assess systematically, might have confounded serum levels and cell counts in patient samples. Further, we could demonstrate a slight decrease of leucocyte numbers directly after initiation of ECMO support, which might indicate that there has been a dilutional effect on early blood samples by priming of the ECMO circuit. A further limitation of our study is that we did not investigate the biological behavior of both the isolated EPC and MSC in mechanistic experiments. Thus, we can only speculate about the potential clinical impact of our findings at this time.

In conclusion, our study results indicate, that ECMO might be associated with an impairment of endogenous mobilization of both EPC and MSC subpopulations, as well as VEGF serum levels in infants with CDH. These results raise the question whether an increase of EPC, MSC or VEGF could beneficially influence the clinical course of CDH patients undergoing ECMO, which needs to be addressed in future studies. Furthermore, future research questions in that regard should also address the necessary amount of stem cells to transplant and the optimal timepoint for stem cell transplantation in the course of ECMO-therapy in CDH. An increase of either EPC or MSC could be accomplished by stimulation of the endogenous mobilization, or by exogeneous transplantation. A potential source of stem cells for therapeutic usage in prenatally diagnosed CDH could be the umbilical cord (42). Currently, there are no clinical trials addressing EPC replacement in pulmonary diseases, however, the therapeutic potential of MSC transplantation in ARDS is currently investigated in phase I and phase II trials (43–45).

## Data Availability Statement

All datasets generated for this study are included in the article/Supplementary Material.

## Ethics Statement

The studies involving human participants were reviewed and approved by Ethics committee of the Medical Faculty Mannheim of the University of Heidelberg. Written informed consent to participate in this study was provided by the participants' legal guardian/next of kin.

## Author Contributions

NR, CP, US, and TS contributed to the concept and design, acquisition, interpretation of data, and drafting of the article. TV, CW, BT, and GB contributed to the interpretation of data and revised the article for important intellectual content. All authors approved the final version of the article.

### Conflict of Interest

The authors declare that the research was conducted in the absence of any commercial or financial relationships that could be construed as a potential conflict of interest.
