# Corrigendum: Endothelial Progenitor and Mesenchymal Stromal Cells in Newborns With Congenital Diaphragmatic Hernia Undergoing Extracorporeal Membrane Oxygenation

**DOI:** 10.3389/fped.2020.00041

**Published:** 2020-02-19

**Authors:** Neysan Rafat, Christian Patry, Ursula Sabet, Tim Viergutz, Christel Weiss, Burkhard Tönshoff, Grietje Beck, Thomas Schaible

**Affiliations:** ^1^Department of Neonatology, University Children's Hospital Mannheim, University of Heidelberg, Mannheim, Germany; ^2^Department of Pediatrics I, University Children's Hospital Heidelberg, Heidelberg, Germany; ^3^Department of Pharmaceutical Sciences, Bahá'í Institute of Higher Education (BIHE), Teheran, Iran; ^4^Department of Anesthesiology and Critical Care Medicine, University Medical Center Mannheim, University of Heidelberg, Mannheim, Germany; ^5^Department for Medical Statistics and Biomathematics, Medical Faculty Mannheim, University of Heidelberg, Mannheim, Germany; ^6^Department of Anesthesiology, Helios Dr. Horst-Schmidt Clinic, Wiesbaden, Germany

**Keywords:** progenitor cells, stem cells, ECMO, congenital diaphragmatic hernia, neonates

In the original article, there was a mistake in Table 1 as published. **The standard deviations in column 1 are incorrect due to a copy/paste error**. The corrected Table 1 appears below.

**Table 1:** Laboratory findings of study subjects.

**Creatinine, blood cell count and c-reactive protein in all study subjects at day 0**			
	**ECMO-dependent**	**ECMO-independent**	**Control**
	**Mean** **±** **SD**	**Mean** **±** **SD**	**Mean** **±** **SD**
Crea [mg/dl]	0.77 ± 0.29	0.73 ± 0.14	0.27 ± 0.00 (*n* = 1)
Hb [g/dl]	14.1 ± 1.36	14.5 ± 1.47	15.9 ± 1.89
Hct [%]	40.4 ± 4.42	40.5 ± 3.65	44.3 ± 6.35
Leukocytes [x10^9^/l]	13.1 ± 5,86	18.4 ± 7.21	10.7 ± 3.84
Platelets [x10^9^/l]	161 ± 51.7^*^	232 ± 65	303 ± 146
CRP [mg/dl]	7.06 ± 15.9	2.21 ± 11.8	0.00 ± 0.00
**Creatinine, blood cell count and c-reactive protein in all study subjects in the disease course**			
	**ECMO-dependent**	**ECMO-independent**	
	**Mean** **±** **SD**	**Mean** **±** **SD**	
Crea [mg/dl]	0.75 ± 0.26^*^	0.46 ± 0.13	
Hb [g/dl]	12.3 ± 0.70^#^	12.7 ± 1.77^#^	
Hct [%]	35.0 ± 1.80^#^	35.7 ± 4.53^#^	
Leukocytes [x10^9^/l]	10.9 ± 4.91	15.1 ± 7.3	
Platelets [x10^9^/l]	111 ± 21.9^*^^#^	302 ± 71.9	
CRP [mg/dl]	24.5 ± 22.3^*^^#^	10.6 ± 16.5^#^	

*Crea, creatinine; CRP, c-reactive protein; Hb, hemoglobin; Hct, hematocrit*.

*Laboratory results of blood cell count, creatinine, and c-reactive protein in all study subjects at day 0 and in the disease course*.

* *Significant difference between ECMO-dependent group and the ECMO-independent group (p < 0.05)*.

# *Significant difference in comparison to the control group (p < 0.05)*.

The authors apologize for this error and state that this does not change the scientific conclusions of the article in any way. The original article has been updated.

