# Supplementary material for: Endothelial Progenitor and Mesenchymal Stromal Cells in Newborns With Congenital Diaphragmatic Hernia Undergoing Extracorporeal Membrane Oxygenation
Source: Front Pediatr. 2019 Nov 22;7:490. doi: 10.3389/fped.2019.00490 (PMC6882772; doi:10.3389/fped.2019.00490)
Supplement: Supplementary file 1 [file Data_Sheet_1.docx]

**Supplemental Material**

**Supplemental Table S1: generic characteristics of the study population**

| comparison of mean age, gender and survival between the study groups | | | | |
| --- | --- | --- | --- | --- |
|  |  | ECMO-dependent | ECMO-independent | healthy control |
|  |  | mean | mean | mean |
| total |  | 18 | 12 | 12 |
| male | | 12 | 5 | 6 |
| female | | 6 | 7 | 6 |
| survivors | | 13 | 11 | 12 |
| non-survivors | | 4 | 1 | 12 |

**Supplemental Table S2: Laboratory findings in the ECMO-dependent group**

| comparison of laboratory findings before and after initiation of ECMO support | | | | |
| --- | --- | --- | --- | --- |
|  |  | ECMO-dependent  day 0 | ECMO-dependent  day 1 | significance |
|  |  | mean ± SD | mean ± SD | p-value |
| creatinin [mg/dl] |  | 0,77 ± 0,29 | 1,00 ± 0,32 | 0,043 |
| hemoglobin [g/l] | | 14,1 ± 1,36 | 12,8 ± 0,93 | 0,003 |
| hematocrit [%] | | 40,4 ± 4,42 | 36,3 ± 2,67 | 0,005 |
| leucocyte count [10^3^ x /cells/µl] | | 13,1 ± 5,86 | 12,0 ± 5,81 | 0,619 |
| platelet count [10^3^ x /cells/µl] | | 161 ± 51,7 | 94,4 ± 26,1 | 0,001 |
| C-reactive protein [mg/dl] | | 7,06 ± 15,9 | 6,56 ± 6,69 | 0,619 |

**Supplemental Table S3: Subpopulations of EPC, MSC, VEGF & Ang2**

Comparison of the counts of different subpopulations of endothelial progenitor cells (EPC) and mesenchymal stem cells (MSC), as well as serum levels of vascular endothelial growth factor (VEGF) and angiopoietin (Ang)2 in all study subjects at day 0 and in the disease course. EPC and MSC counts are expressed as percentage of total peripheral blood mononuclear cells. *significant difference between the ECMO-dependent group and the ECMO-independent group (p<0,05); #significant difference in comparison to the control group (p<0,05).

| EPC & MSC subpopulations, VEGF and Ang2 counts in all study subjects at day 0 | | | | | | | | | |
| --- | --- | --- | --- | --- | --- | --- | --- | --- | --- |
|  |  | ECMO-dependent | | ECMO-independent | | | healthy control | |  |
|  |  | mean ± SD | | mean ± SD | | | mean ± SD | |  |
|  |  |  | |  | | |  | |  |
| EPC-CD45^dim^/CD34^+^ [%] | | 0,86 ± 0,41 * # | | 0,33 ± 0,14 | | | 0,12 ± 0,10 | |  |
| EPC-CD45^dim^/CD34^+^/CD133^+^ [%] | | 0,68 ± 0,30 * # | | 0,26 ± 0,11 | | | 0,10 ± 0,09 | |  |
| EPC-CD45^dim^/CD34^+^/CD133^+^/CD31^+^ [%] | | 0,80 ± 0,28 * # | | 0,11 ± 0,10 | | | 0,09 ± 0,08 | |  |
| MSC-CD34^-^/CD73^+^/CD29^+^ [%] | | 11,4 ± 4,09 | | 6,32 ± 4,30 | | | 3,54 ± 6,78 | |  |
| MSC-CD34^-^/CD73^+^/CD29^+^/CD90^+^ [%] | | 6,60 ± 4,98 | | 4,80 ± 3,75 # | | | 0,17 ± 0,32 | |  |
| MSC-CD34^-^/CD73^+^/CD90^+^ [%] | | 7,54 ± 4,72 | | 5,34 ± 3,57 # | | | 0,34 ± 0,57 | |  |
| MSC-CD34^-^/CD29^+^/CD90^+^ [%] | | 16 ± 9,82 | | 19 ± 10,8 # | | | 6,01 ± 11,2 | |  |
| VEGF [pg/ml] | | 347 ± 198 | | 458 ± 277 | | | 568 ± 321 | |  |
| Ang2 [pg/ml] | | 6339 ± 2008 | | 6495 ± 1846 | | | 6818 ± 2220 | |  |
|  | |  |  | | |  | | |  |
| EPC & MSC subpopulations, VEGF and Ang2 counts in all study subjects in the disease course | | | | | | | | | |
|  |  | ECMO-dependent | | | ECMO-independent | | |  | |
|  |  | mean ± SD | | | mean ± SD | | |  | |
|  |  |  | | |  | | |  | |
| EPC-CD45^dim^/CD34^+^ [%] | | 0,61 ± 0,41 * # | | | 0,30 ± 0,14 # | | |  | |
| EPC-CD45^dim^/CD34^+^/CD133^+^ [%] | | 0,48 ± 0,30 * # | | | 0,23 ± 0,11 # | | |  | |
| EPC-CD45^dim^/CD34^+^/CD133^+^/CD31^+^ [%] | | 0,49 ± 0,28 * # | | | 0,18 ± 0,10 | | |  | |
| MSC-CD34^-^/CD73^+^/CD29^+^ [%] | | 8,08 ± 4,09 # | | | 9,99 ± 4,30 # | | |  | |
| MSC-CD34^-^/CD73^+^/CD29^+^/CD90^+^ [%] | | 4,89 ± 4,98 # | | | 7,61 ± 3,75 # | | |  | |
| MSC-CD34^-^/CD73^+^/CD90^+^ [%] | | 6,19 ± 4,72 # | | | 8,86 ± 3,57 # | | |  | |
| MSC-CD34^-^/CD29^+^/CD90^+^ [%] | | 12,7 ± 9,82 # | | | 21,0 ± 10,8 # | | |  | |
| VEGF [pg/ml] | | 218 ± 198 * # | | | 539 ± 277 | | |  | |
| Ang2 [pg/ml] | | 10053 ± 2008 * # | | | 7602 ± 1846 | | |  | |

**Supplemental Table S4:** **Mobilizing factors of EPC and MSC**

Mean serum levels of vascular endothelial growth factor (VEGF) and angiopoietin (Ang)2 at different time points of blood sample acquisition (blood samples in the ECMO-dependent group at day 0, day 1 and day 3; blood samples in the ECMO-independent group at day 0, day 3 and day 7) in the ECMO-dependent group and the ECMO-independent group. *significant difference compared to the first blood sample (p<0,05).

|  | ECMO-dependent | | | |  |
| --- | --- | --- | --- | --- | --- |
|  |  |  |  |  | |
|  |  | day 0 | day 1 | day 3 | |
|  |  |  |  |  | |
| VEGF | mean [pg/ml] | 347 | 28,1 * | 34 * | |
|  | +/- SD | 283 | 57,1 | 36,9 | |
|  |  |  |  |  | |
| Ang2 | mean [pg/ml] | 6339 | 11081 * | 10756 * | |
|  | +/- SD | 3618 | 2854 | 2261 | |
|  | ECMO-independent | | | |  |
|  |  |  |  |  | |
|  |  | day 0 | day 3 | day 7 | |
|  |  |  |  |  | |
| VEGF | mean [pg/ml] | 458 | 598 | 536 | |
|  | +/- SD | 280 | 456 | 248 | |
|  |  |  |  |  | |
| Ang2 | mean [pg/ml] | 6495 | 7812 | 7109 | |
|  | +/- SD | 2916 | 3692 | 2354 | |

**Supplemental Table S5:** **Clinical information for ECMO-dependent CDH patients**

Additional clinical information about the ECMO-dependent group. Position of stomach, parts of the intestines and liver is shown in relation to the diaphragm. Localization refers to left- or right sided herniation.

| WGA | umb. art. pH | pos. of stomach | pos. of intestines | pos. of liver | loc. of liver | LHR | rel.  LHR | mean lung volume | rel.  lung volume |
| --- | --- | --- | --- | --- | --- | --- | --- | --- | --- |
| 35+0 | 7,33 | ND | ND | down | left | 1,60 | 75,34 | ND | 25 |
| 39+2 | 7,28 | ND | ND | ND | right | ND | ND | ND | ND |
| 36+2 | 7,37 | ND | ND | up | left | 1,20 | 26,07 | ND | 24 |
| 36+0 | 7,20 | thorax | thorax | up | left | 0,60 | 17,57 | 24,14 | 24 |
| 37+0 | 7,33 | thorax | thorax | up | left | 1,40 | 32,97 | 30,57 | 18 |
| 36+1 | 6,95 | ND | ND | ND | left | ND | ND | ND | ND |
| 35+5 | 7,34 | thorax | thorax | up | left | 1,60 | 37,55 | 30,71 | 21 |
| 33+5 | 7,17 | ND | ND | up | left | 0,69 | 15,22 | 33 | 20 |
| 39+0 | 7,31 | ND | ND | up | left | > 1,4 | > 29,19 | ND | 30 |
| 37+5 | 7,34 | ND | ND | up | left | 1,40 | 33,33 | 29,14 | 35 |
| 38+3 | 7,10 | thorax | thorax | up | left | 1,50 | 36,72 | 29,14 | 37 |
| 38+0 | 7,38 | thorax | thorax | up | left | 1,80 | 38,16 | 32,71 | 22 |
| 37+2 | 7,33 | thorax | thorax | up | left | 1,10 | 28,31 | ND | 20 |
| 37+2 | ND | thorax | thorax | up | left | 0,90 | 20,45 | 32,14 | 20 |
| 37+1 | 7,30 | ND | ND | up | left | 1,70 | 38,74 | ND | 32 |

*LHR*, lung to head ratio; *loc.,* localization; *ND*, no data available; *pos.,* position; *rel.,* relative*; umb. art.,* umbilical artery; *WGA*, weeks gestational age

**Supplemental Table S6:** **Clinical information for ECMO-independent CDH patients**

Additional clinical information about the ECMO-independent group. Position of stomach, parts of the intestines and liver is shown in relation to the diaphragm. Localization refers to left- or right sided herniation.

| WGA | umb. art. pH | pos. of stomach | pos. of intestines | pos. of liver | loc. of liver | LHR | rel.  LHR | mean lung volume | rel.  lung volume |
| --- | --- | --- | --- | --- | --- | --- | --- | --- | --- |
| 38+3 | 7,20 | thorax | thorax | left | down | 2,00 | 41,98 | 38 | 30 |
| 39+5 | 7,28 | thorax | abd | left | up | 2,00 | 41,64 | 39,71 | 36 |
| 38+0 | 7,31 | ND | ND | left | down | 1,90 | 40,34 | ND | 25 |
| 37+6 | 7,30 | thorax | thorax | left | up | 1,80 | 38,45 | 36 | 33 |
| 38+0 | 7,36 | thorax | thorax | left | up | 1,73 | 43,88 | 31,71 | 28 |
| 37+0 | 7,28 | ND | ND | left | ND | ND | ND | ND | ND |
| 38+3 | 7,31 | abd. | thorax | left | down | 1,80 | 38,79 | 35,14 | ND |
| 39+0 | 7,35 | thorax | thorax | left | down | 1,60 | 34,05 | 33,29 | ND |
| 38+2 | 7,41 | ND | ND | left | ND | ND | ND | ND | ND |
| 37+3 | 7,37 | thorax | thorax | left | up | 1,75 | 38,52 | 33,86 | 24 |
| 38+4 | 7,35 | abd. | ND | left | down | 2,00 | 41,77 | ND | 35 |
| 38+1 | 7,31 | thorax | thorax | left | down | 2,40 | 55,74 | 31,14 | 48 |

*abd*., abdominal; *LHR*, lung to head ratio; *loc.,* localization; *ND*, no data available; *pos.,* position; *rel.,* relative*; umb. art.,* umbilical artery; *WGA*, weeks gestational age

**Supplemental Figure S1**


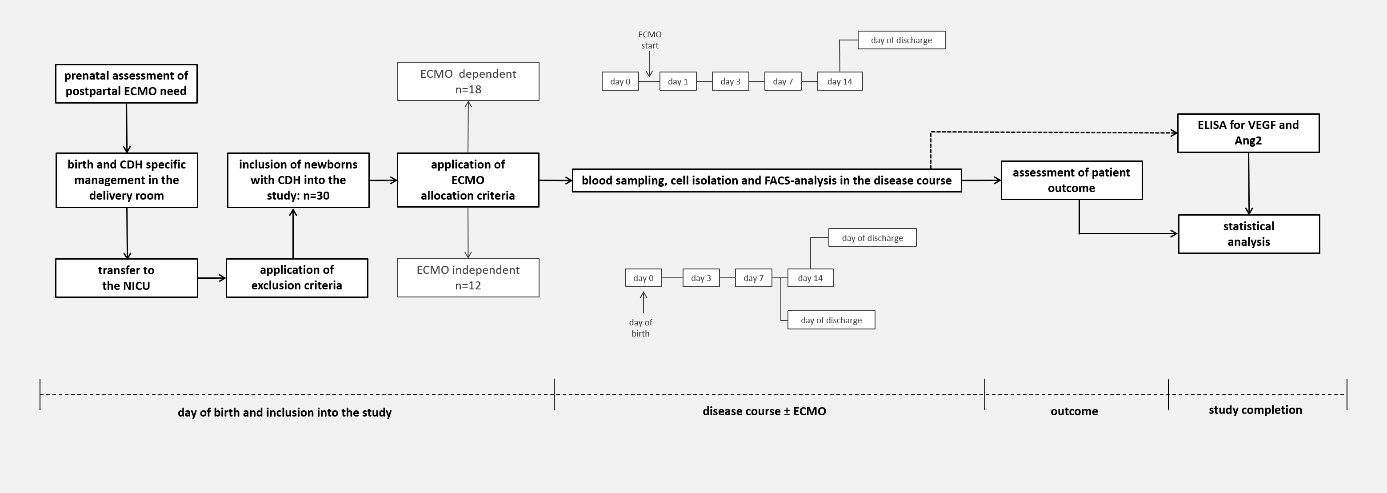


**Supplemental Figure S1.** Flow chart visualizing the experimental design, timing of blood sampling, patient recruitment and assessment of patient outcome of the study.

**Supplemental Figure S2**

**Supplemental Figure S2. Correlation of endothelial progenitor cells with relative lung-to-head ratio and relative lung volume.** The subpopulation of endothelial progenitor cells (EPC) CD34^+^/45^dim^/133^+^ correlates significantly with the relative lung-to-head ratio (rLHR) [r: -0,58; p=0,0044] **(A)** and the relative lung volume (rel. LuVol) [r: -0,49; p=0,0217] (**B)**.

**Supplemental Information on Materials**

The following Antibodies were used to detect cell surface antigens in our study:

PE-conjugated anti-human CD133 monoclonal antibody (Miltenyi Biotec, Bergisch-Gladbach, Germany)

FITC-conjugated anti-human CD34 monoclonal antibody (BD Biosciences, Heidelberg, Germany)

PerCP-conjugated anti-human CD45 monoclonal antibody (Miltenyi Biotec, Bergisch-Gladbach, Germany)

APC-conjugated anti-human CD31 monoclonal antibody (Miltenyi Biotec, Bergisch-Gladbach, Germany)

PE-conjugated anti-human CD90 monoclonal antibody (Miltenyi Biotec, Bergisch-Gladbach, Germany)

FITC-conjugated anti-human CD29 monoclonal antibody (BD Biosciences, Heidelberg, Germany)

PerCP-conjugated anti-human CD34 monoclonal antibody (BD Biosciences, Heidelberg, Germany)

APC-conjugated anti-human CD73 monoclonal antibody (BD Biosciences, Heidelberg, Germany)
